# Silicon‐Mediated Mitigation of Salt Stress in Maize Plants

**DOI:** 10.1002/pei3.70073

**Published:** 2025-07-28

**Authors:** Moniba Zahid Mahmood, Muhammad Shahzad, Muhammad Nauman, Arshad Mehmood Abbasi, Michael Reichelt, Axel Mithöfer

**Affiliations:** ^1^ Research Group Plant Defense Physiology Max Planck Institute for Chemical Ecology Jena Germany; ^2^ Department of Environmental Sciences COMSATS University Islamabad, Abbottabad Campus Abbottabad Pakistan; ^3^ Department of Biochemistry Max Planck Institute for Chemical Ecology Jena Germany

**Keywords:** phytohormones, salt stress, silicon, *Zea mays*

## Abstract

High soil salinity affects plant growth, yield, and water use efficiency, leading to drought and ion toxicity. Silicon (Si), a crucial element in soil, can mitigate such stress. Si neutralizes harmful impacts, reduces Na^+^ uptake, and promotes plant growth. It benefits higher plants like grasses and cultivated crops. However, its role in maize cultivars is rarely reported. The present study aimed to evaluate the impact of exogenous Si application on maize plant growth, physiology, gene activation, and phytohormonal regulation under salinity stress. Therefore, a hydroponic experiment was conducted to study the impact of salt (100 mM NaCl) on two different maize varieties, the salt‐sensitive Jalal and the salt‐tolerant Iqbal, along with and without Si enrichment in pots or Si foliar spray. Our findings revealed that various phenotypical growth parameters as well as physiological parameters were significantly affected due to salt stress. However, the presence of Si mitigated the stress responses in both varieties. Moreover, we found that Si application reduced the NaCl‐induced effects on abscisic acid and jasmonates in both varieties. Based on our findings, we concluded that Si application may lead to a reduction of NaCl‐mediated salt stress on maize plants and help the plant to grow better.

## Graphical abstract

Silicon application on maize plants changes growth, physiology, gene activation and phytohormonal regulation under salinity stress. Thereby, silicon attenuates the salt stress and protects the maize plants.

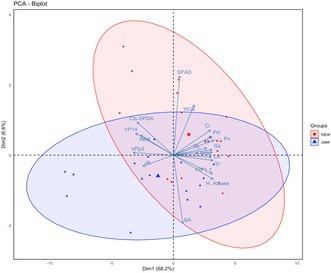

## 1 Introduction

In today's world, soil salinization is one of the biggest environmental problems. Salinization has affected up to 20% of the world's arable land and 50% of all irrigated land (Yin et al. 2019). Unsuitable irrigation methods severely affect the growth and development of plant species (Shrivastava and Kumar 2015). Saline environments disrupt plant processes like protein production and photosynthesis, leading to poor seed germination, reduced productivity, and detrimental effects on plant physiology (Butcher et al. 2016), biomass production (Basyuni et al. 2019), mineral ion uptake, and biochemical damage (Yan et al. 2018; Shahid et al. 2020). The excess of salt can also trigger oxidative stress, reducing the ability of plants to scavenge ROS, which damages cell membranes and biomolecules, contributing to overall growth inhibition (Zhu and Gong 2014; Rehman et al. 2019).

In most salt‐affected environments, NaCl is the predominant salt, and its main detrimental effect on non‐resistant plants is growth inhibition by osmotic balance disruption in the plant cells, reducing water uptake and nutrient uptake (Forieri et al. 2016). Na^+^ ion in excess can affect plant health; its high concentration increases cytosolic Ca^2+^ concentration and subsequently activates anion channels localized in the plasma membrane (Lee and Luan 2012), causing guard cell depolarization, potassium efflux, loss of guard cell turgor and volume, and eventually leads to stomatal closure (Hasanuzzaman and Fujita 2022).

Plants adapt to abiotic stresses by changing morphological and developmental patterns, physiological and biochemical processes, and metabolic adjustments. These include the accumulation of organic solutes, the protection of cellular machinery, ionic homeostasis, scavenging free radicals, the expression of proteins, gene up‐regulation, and the induction of phytohormones (Zarate et al. 2007). Phytohormones regulate growth and development responses under ambient and stressful conditions. They control physiological processes and help plants to adapt to abiotic stress (Iqbal et al. 2014). Recent studies have focused on understanding plant salt tolerance and its consequences and on controlling ionic homeostasis and osmotic stress (Hussain et al. 2021). Phytohormones are widely acknowledged as crucial in modulating biochemical events and physiological processes under salt stress. Endogenous levels of these compounds can predict plant tolerance or susceptibility (Hussain et al. 2024). Understanding the role of individual phytohormones could provide crucial information on saline soil adaptation. Salicylic acid (SA) is known to induce systemic acquired resistance against pathogens, but recently its role has also been emphasized in abiotic stress tolerance, like drought and salinity (Hara et al. 2012). Jasmonic acid (JA) also stimulates defense‐related signaling in response to herbivory, and it is also important in the accumulation of abscisic acid (ABA) during osmotic stress (Han et al. 2013; de Ollas et al. 2013). ABA plays an important role in seed development, dormancy, and response to environmental stresses. It regulates plant water status and growth through guard cells and gene induction. It increases due to salt stress and is a crucial stress hormone regulating salt‐responsive genes under salinity in plants.

In the last decade, studies have revealed the mitigating role of Si under various biotic (plant diseases and pests) and abiotic stresses (heavy metals, drought, and salinity) on crop plants (Lee and Luan 2012). Si is the second most abundant element in the earth's crust and is believed to be a principal component of soil (Zhu and Gong 2014). Plants growing in terrestrial environments have a concentration of Si ranging from 1% to 10% or even higher (Keller et al. 2015). However, Si is not considered an essential plant nutrient but plays a major role in plant growth, especially under stress (Adrees et al. 2015). Si has been widely reported to alleviate salt stress by reducing Na^+^ uptake and/or Na^+^ transport to the shoot. For example, adding Si reduces Na^+^ accumulation in rice shoots, which is attributed to a reduction in Na^+^ transport across the bypass flow caused by silicon deposition (Gong et al. 2006). Tahir et al. (2021) found that Si addition decreased Na^+^ uptake and increased K^+^ uptake in wheat plants under salt stress. In addition to ion toxicity, high salt concentrations in the soil also lead to osmotic stress, which limits plant water uptake by the roots. Chen et al. (2014) reported that the alleviating effect of silicon on salt‐induced osmotic stress is more pronounced than salt‐induced ion toxicity in wheat under salt stress (Hurtado et al. 2020). Si can alleviate adverse effects of drought and salt stresses on *Glycyrrhiza uralensis* seedlings by regulating nitrogen metabolism and triggering secondary metabolite accumulation (Chen et al. 2025). Si also increased the salt tolerance of cucumber mainly by improving root water uptake rather than by reducing Na^+^ uptake or transport (Ahammed and Yang 2021; Ismail et al. 2022), and promoting plant growth through improved uptake of mineral nutrients and water (Hernández‐Salinas et al. 2022; Sayed et al. 2022). According to Karvar et al. (2023) foliar spraying with K_2_SiO_3_ reduced drought stress effects in sweet corn plants and improved physiological traits and crop yield. Thus, research has revealed the advantageous effects of Si under salt stress on higher plants, particularly benefiting grasses and various cultivated crops such as rice, tomato, wheat, and cucumber (Alzahrani et al. 2018; Zhu et al. 2020). However, its role and responsible mechanisms in alleviating salt stress in maize cultivars are rarely reported in the literature. In particular, the role of Si in phytohormonal regulation at the transcriptomic level during salinity stress has yet to be elucidated.

Maize ( *Zea mays* L.) is one of the most important food crops used by both humans and animals. It has been reported that maize is grown worldwide in an area of more than 142 million hectares, accounting for one‐third of the global grain yield (Erenstein et al. 2021). As a salt‐sensitive crop, maize experiences detrimental effects on its morphology, biochemistry, and physiological mechanisms, leading to reduced seed germination, biomass, photosynthesis, and mineral nutrient accumulation (Kamran et al. 2019; Naveed et al. 2020).

Here, two different maize varieties were under investigation, the salt‐tolerant Iqbal and the salt‐sensitive Jalal (Mahmood et al. 2024). The study aimed to assess the effects of exogenous Si application on vegetative maize plant growth parameters, physiology, and phytohormonal regulation under salinity stress. We additionally analyzed phytohormones (ABA, JA, and SA) as well as the expression of four salt stress‐related genes. These genes play a role in ion homeostasis, water balance, hormonal signaling, and subcellular pH, which are all important for mitigating the stress imposed by salinity. The study provides useful information for corn production in salt‐affected areas challenged by soil salinization.

## 2 Materials and Methods

### 2.1 Experimental Design and Growth Conditions

Seeds of two maize ( *Zea mays* L.) varieties (Iqbal and Jalal) were obtained from the Cereal Crops Research Institute (CCRI), Pirsabak, Nowshera, Pakistan. The seeds were soaked in 0.5 mM calcium sulfate overnight. Seedlings were grown in quartz sand in hydroponic culture in the greenhouse at the Max Planck Institute for Chemical Ecology, Jena, Germany. The growth of seedlings was carried out for 10–12 h under ambient light at ~28°C with 65%–70% humidity. At the two‐leaf stage, uniform seedlings were uprooted and transplanted into rock wool and transferred to small pots in hydroponic chambers. After 1 week of seedling development in hydroponic culture, thinning was carried out, and only one healthy seedling was kept in each pot per replication of all the treatments. Afterward, a salinity level of 100 mM was provided using NaCl salt. One week later, when salinity toxicity symptoms appeared on plants, 1 mM calcium silicate (Ca_2_SiO_4_) was added with different treatments in the nutrient solution. The experimental treatments were organized in a complete randomization with six replications. Treatments include Control; (C), 1 mM Ca_2_SiO_4_; (Si), 1 mM Foliar Ca_2_SiO_4_; (FSi), 100 mM NaCl; (NaCl), 1 mM Ca_2_SiO_4_ + 100 mM NaCl; (Si + NaCl) and 1 mM Foliar Ca_2_SiO_4_ + 100 mM NaCl; (FSi + NaCl). The pH of the nutrient media was maintained at 6.5 ± 0.5. To prevent nutrient deficits, the nutrition solution was replaced twice weekly.

### 2.2 Plant Harvesting and Morphological Characteristics of Maize Varieties

After 21 days of salinity stress and Si supply, maize plants were harvested, washed thoroughly, tissue dried, and transferred to the laboratory to determine growth parameters. The total leaf area, leaf length, shoot height, and root length were analyzed using ImageJ software version 23.0. Fresh and dry weight of shoots and roots were determined using a digital weighing balance. Afterwards, the dried samples were stored in a cool, dry place for further use.

### 2.3 Physiological Characteristics of Maize Cultivars

Water use efficiency (WUE) was determined by dividing the dry weight of the whole plant by the cumulative amount of water transpired during the specific growing stage (Zhang et al. 2015).

The membrane stability index (MSI) was measured according to (Sairam and Saxena 2000). Fresh leaves (100 mg) were treated at 40°C for a half‐hour in distilled water (10 mL) in a water bath, and electrical conductivity (EC) was noted (C1). Subsequently, the samples were heated at 100°C for 10 min, and the EC was indicated as C2 (Kumar et al. 2015). The noted measurements were utilized to measure the MSI as below:

$$\mathrm{MSI}\%=\left[1-\left(C1/\text{C2}\right)\times 100\right]\;\mathrm{RWC}\%=\left(\mathrm{FW}-\mathrm{DW}\right)/\left(\mathrm{TW}-\mathrm{DW}\right)\times 100$$

The chlorophyll content of maize plants' leaves was determined using a CCM 200+ meter (Almansoori et al. 2021).

### 2.4 Gas Exchange Traits in the Leaves of Maize Cultivars

Gaseous exchange measurements (net transpiration rate, stomatal conductance, net photosynthesis, and internal CO_2_ concentration) were recorded between 9.00 a.m. and 11.00 a.m. by placing the youngest upper leaves of each plant in the leaf cuvette from each treatment by using a portable infrared gas analyzer (IRGA; LC pro‐ADC, Hoddesdon, UK). IRGA was set at a 0–3000 ppm CO_2_ range, the temperature inside the leaf chamber was +15°C to −10°C, and the leaf chamber window area was 6.25 cm^2^.

### 2.5 Phytohormone Analysis

Leaves of maize plants treated with and without Si were detached and immediately frozen in liquid nitrogen, crushed with the help of a Geno Grinder, and approximately 250–3000 mg of dry leaf material was extracted with 1.5 mL methanol containing 60 ng D4‐SA (Santa Cruz Biotechnology, USA), 60 ng D6‐JA (HPC Standards GmbH, Germany), 60 ng D6‐abscisic acid (ABA) (Santa Cruz Biotechnology), and 12 ng D6‐jasmonoyl isoleucine (JA‐Ile) (HPC Standards GmbH) as internal standards. Then, samples were processed as previously described (Heyer et al. 2018). The following phytohormone separation and quantification were performed by high‐performance liquid chromatography coupled to tandem mass spectrometry (HPLC‐MS/MS) (Agilent 1260; Agilent Technologies, Santa Clara, CA, USA; API 6500, SCIEX, Darmstadt, Germany) with a Zorbax Eclipse XDB‐C18 column (50 × 4.6 mm, 1.8 μm; Agilent Technologies). The detailed MS protocol in negative ionization mode has been described previously (Heyer et al. 2018).

### 2.6 Gene Expression Assays, RT‐qPCR

For gene expression analysis, RNA was extracted from leaves. Total RNA extraction and purification were performed using the Total RNA kit (50) (Sigma‐Aldrich) and DNase I (Zymo Research) kit according to manufacturer recommendations. RNA purity, quantity, and quality were assessed using a spectrophotometer (NanoDrop, 2000c; Thermo Scientific). One microgram of RNA was used for cDNA synthesis with the Revert Aid First Strand cDNA Synthesis Kit (Thermo Scientific) using oligo (dT)18 primers according to the manufacturer's instructions. The RT‐qPCR reaction was set as follows: 10 μL SYB green Master Mix (Thermo Scientific), 0.5 μL forward primer (10 μM), 0.5 μL reverse primer (10 μM), 5 μL cDNA, and 4 μL nuclease‐free water using the following program: initial denaturation (95°C, 60 s, 1×), denaturation (95°C, 15 s), extension (60°C, 30 s) with 40 cycles, and an additional melting curve (60°C–95°C, 1×). The expression of the following *Z. mays* genes was analyzed: *Zea mays plasma membrane intrinsic protein* (LOC542619), *vacuolar ATPase subunit H* (LOC100280574), *vacuolar proton pump 3* (LOC542327), and *viviparous 14* (LOC732819). All these genes are somehow linked to ABA and stomatal opening and closure. *PM H* ^+^ *‐ATPase* encodes a proton pump that is crucial in ion transport across the plasma membrane, regulating cell turgor pressure and pH homeostasis (Haruta et al. 2015); *vacuolar proton pump* (*VPp3*) genes encode proton pumps that play a crucial role in plant cell ion homeostasis and pH regulation (Mallikarjuna et al. 2020). Both are essential genes in plant homeostasis, preventing salt toxicity and maintaining cellular and osmotic balance. The *ZmPIP1:1* gene encodes an aquaporin protein involved in water transport across cell membranes (Bienert et al. 2018); *Viviparous‐14* (*VP14*) is a gene encoding a key biosynthetic gene in ABA synthesis (Messing et al. 2010). Actin was used as a reference gene (actin 1 [LOC100282267]). The primer list and more information are presented in Table 1 and Supporting Information. The normalized fold gene expression was calculated with the help of Ct value (2^−ΔΔCt^) (Pfaffl 2001).

**TABLE 1:** Primers used for qPCR analyses.

Gene	Abbreviation	Sequence (5′ → 3′)	bp
*Zea mays* plasma membrane intrinsic protein	*Zm*PIP1‐1 F	CGGAGGCGTGAACTGTAGAT	72
	*Zm*PIP1‐1 R	ATGGCTAGAGGCAACCAACG	
Viviparous 14	*Zm*VP14 F	TGTTGTCACCCAGTCCAGTG	141
	*Zm*VP14 R	CCGATAGCCACAGGGAACAC	
Vacuolar proton pump 3	VPp3 F	GCACGGTGAACTGCTGTAGA	118
	VPp3 R	AGAAATGAGTAGCGGTGGGG	
Plasma membrane H^+^‐ATPase	H^+^‐ATPase F	CCGTCTCGCCCTCATCTAA	111
	H^+^‐ATPase R	TGGAAGAAGGATCGGGAGAC	
Reference gene	Actin F	ATCCGTCATAGCAACCCGC	72
	Actin R	GCGAGGAAAAATAGGGGAGC	

### 2.7 Statistical Analysis

Data were first verified for normality by means of the Shapiro–Wilk test and for homogeneity using Levene's test. If both assumptions were met, the data were further analyzed using two‐way analysis of variance (ANOVA) and Tukey's test. Tests were performed using the univariate general linear model, which yields significant differences between means that were distributed normally and compared at significance level *p* ≤ 0.05. These differences were labeled using small letters positioned at the top of each bar. The data are presented as the means ± standard errors (SE). SPSS, the Statistical Package for the Social Sciences, version 23.0, was used to analyze the data. R studio software (2024.12.0‐467) was utilized for principal component analysis (PCA), generating biplots with the first two components (PC1 and PC2) explaining the most significant dataset variations.

## 3 Results

### 3.1 Effect of Ca_2_SiO_4_ on the Morphology of Salt‐Stressed Maize Varieties

**FIGURE 1:**
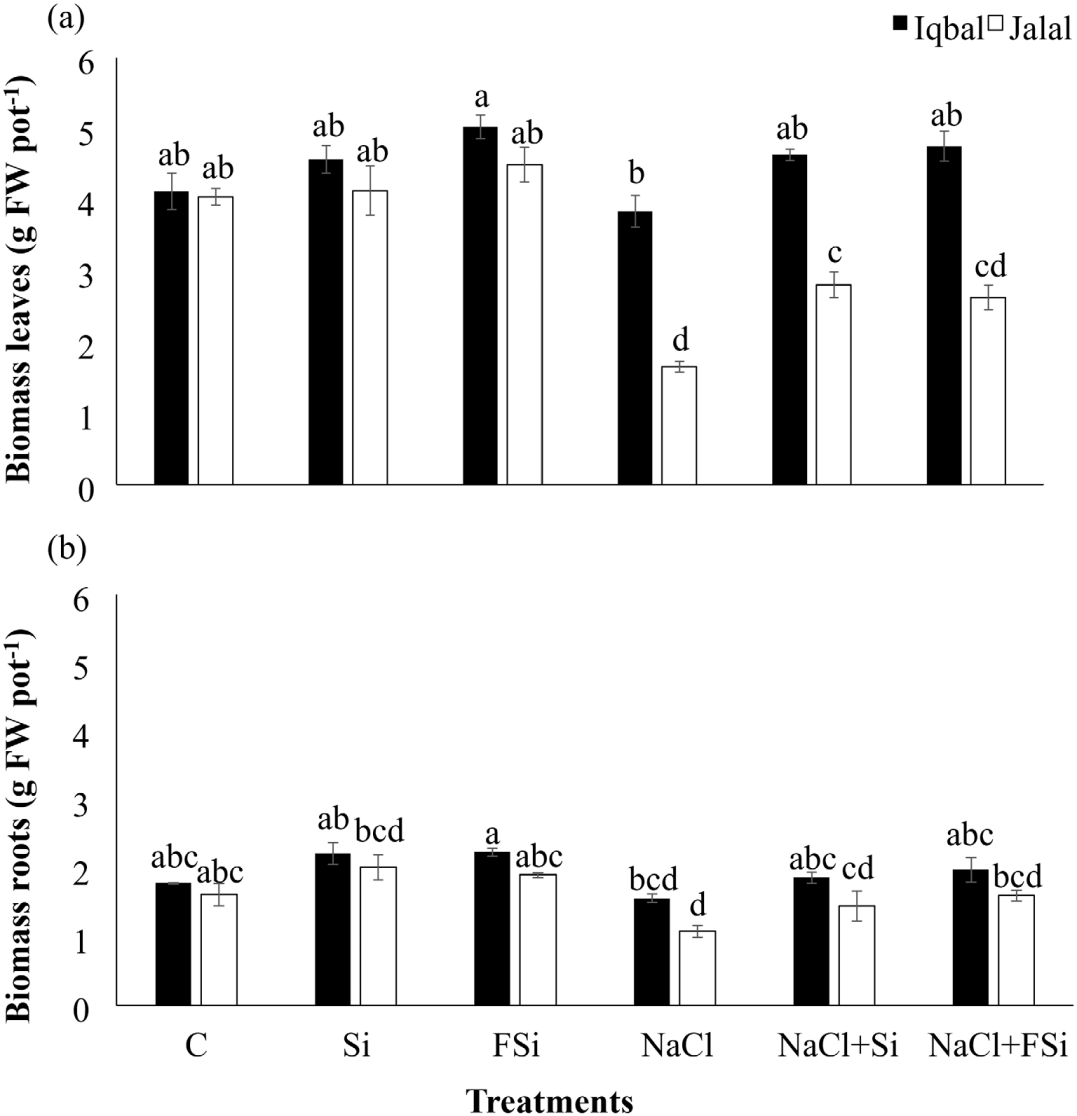
Effect of Ca_2_SiO_4_ on the production of fresh and dry biomass (g pot^−1^) under salt stress. (a) Fresh weight leaves, (b) fresh weight roots in two different maize varieties (Iqbal and Jalal) under NaCl stress. The result was revealed in means, standard error (±), difference between the treatments (*p* ≤ 0.05, *n* ≥ 3) as indicated by different letters. (C): Control, (Si): 1 mM Ca_2_SiO_4_, (FSi): Foliar 1 mM Ca_2_SiO_4_, (NaCl) = 100 mM NaCl; (NaCl + Si) = 100 mM NaCl + 1 mM Ca_2_SiO_4_, (NaCl + FSi) = 100 mM NaCl + Foliar 1 mM Ca_2_SiO_4_.

In comparison to the control, the Jalal variety showed a 59.03% decline in FW when the plant was treated with NaCl, while leaf FW in Iqbal was hardly affected (Figure 1). Treatments with Si alone did not affect the plants. Upon NaCl stress, both pot and foliar Si treatment mitigated the salt‐induced stress response on the leaves FW (Figure 1a). This effect was stronger and significant (*p* < 0.05) in Jalal rather than in Iqbal leaves (Figure 1a). In roots, the FW changes were similar but less pronounced (Figure 1b). Compared with controls, again the Jalal variety showed a significant decline in FW when the plant was treated with NaCl (*p* < 0.05), in contrast to Iqbal. Any treatment with Si caused an increase in root FW in both varieties (Figure 1b).

**FIGURE 2:**
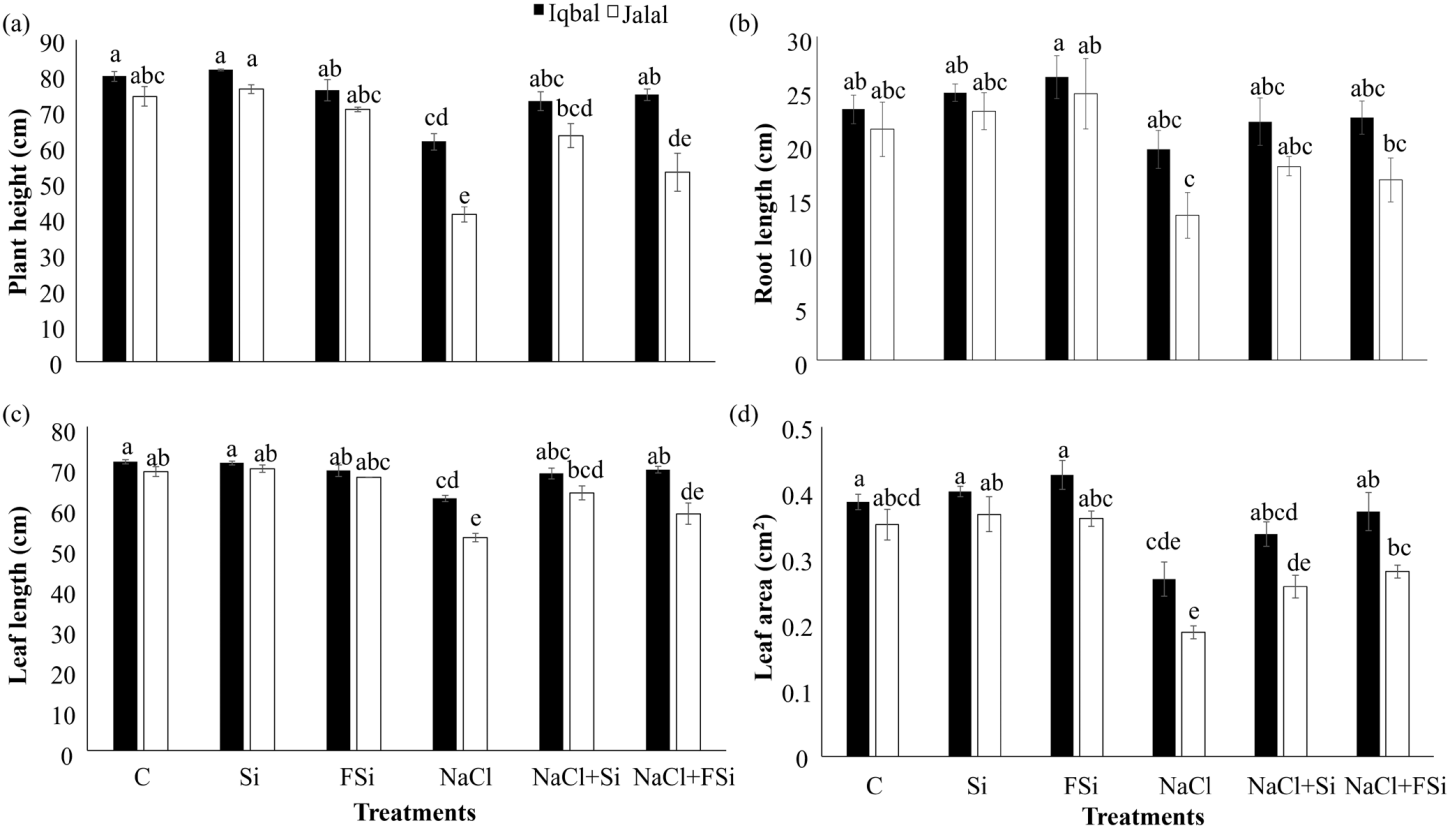
Effect of Ca_2_SiO_4_ on the growth of maize under salt stress. (a) Plant height (cm), (b) root length (cm), (c) leaf length (cm), (d) leaf area (cm^2^) in two different maize varieties (Iqbal and Jalal) under NaCl stress. The result was revealed in means, standard error (±), difference between the treatments (*p* ≤ 0.05, *n* ≥ 3) as indicated by different letters. (C): Control, (Si): 1 mM Ca_2_SiO_4_, (FSi): Foliar 1 mM Ca_2_SiO_4_, (NaCl) = 100 mM NaCl; (NaCl + Si) = 100 mM NaCl + 1 mM Ca_2_SiO_4_, (NaCl + FSi) = 100 mM NaCl + foliar 1 mM Ca_2_SiO_4_.

Further, phenotypical data related to other growth parameters, including plant height, root length, leaf length, and leaf area, were monitored and compared to determine the effect of Ca_2_SiO_4_ on the growth of maize varieties under salt‐stress conditions (Figure 2). Treatment with 100 mM NaCl significantly decreased (*p* < 0.05) shoot height, leaf length, and leaf area in Jalal and Iqbal. This effect was mitigated by Si treatments (Figure 2). Concerning root length, the same but non‐significant trends were found (Figure 2).

### 3.2 Effect of Salt Stress on Physiological Features of Salt Stressed *Z. mays* Varieties

The results regarding chlorophyll contents, MSI, and WUE of both maize varieties grown under saline stress with or without external application of Ca_2_SiO_4_ was analyzed as well (Table 2). The data for chlorophyll in Iqbal and Jalal showed that NaCl stress decreased chlorophyll content in both maize varieties. Upon Si treatments, SPAD value increased again (Table 2). The same was found for MSI and root to shoot ratio, while the data for WUE did not show significant changes (Table 2).

**TABLE 2:** Influence of Ca_2_SiO_4_ application on: membrane stability index %, SPAD unit, water use efficiency (μmol CO_2_ mmol^−1^), and root/shoot ratio in two different maize varieties (Iqbal and Jalal) under NaCl stress.

		Control	Si	Foliar Si	NaCl	NaCl + Si	NaCl + foliar Si
MSI %	Iqbal	81.86 ± 2.89ab	87.02 ± 4.04ab	85.94 ± 2.42a	68.74 ± 2.59b	77.78 ± 4.16ab	75.52 ± 3.27ab
	Jalal	81.17 ± 3.49ab	85.44 ± 0.38ab	81.77 ± 3.45ab	54.36 ± 1.23d	65.95 ± 3.28c	73.77 ± 2.49 cd
SPAD	Iqbal	43.94 ± 1.14ab	45.84 ± 1.41ab	48.38 ± 1.00a	28.96 ± 1.17f	39.92 ± 1.08bcde	35.08 ± 0.97cdef
	Jalal	42.88 ± 1.47ab	40.32 ± 1.00bcd	44.44 ± 0.35abc	26.16 ± 1.80f	34.88 ± 1.47def	34.58 ± 1.46ef
WUE	Iqbal	5.64 ± 0.04ab	5.59 ± 0.41ab	5.75 ± 0.15ab	5.76 ± 0.29ab	6.07 ± 0.36a	5.92 ± 0.22a
	Jalal	5.14 ± 0.27ab	5.74 ± 0.11ab	5.83 ± 0.23ab	4.62 ± 0.28b	5.46 ± 0.10ab	5.37 ± 0.34ab
Root/shoot	Iqbal	1.61 ± 0.25a	1.46 ± 0.43abcd	1.40 ± 0.40abcd	0.65 ± 0.06d	1.05 ± 0.13bcd	1.68 ± 0.12abcd
	Jalal	1.58 ± 0.18ab	1.51 ± 0.07abcd	1.38 ± 0.26abcd	0.76 ± 0.07 cd	1.19 ± 0.21bcd	1.32 ± 0.12abc

*Note:* The result was revealed in means, standard error (±), difference between the treatments (*p* ≤ 0.05, *n* ≥ 3) as indicated by different letters. (C): Control, (Si): 1 mM Ca_2_SiO_4_, (FSi): Foliar 1 mM Ca_2_SiO_4_, (NaCl) = 100 mM NaCl; (NaCl + Si) = 100 mM NaCl + 1 mM Ca_2_SiO_4_, (NaCl + FSi) = 100 mM NaCl + foliar 1 mM Ca_2_SiO_4_.

**FIGURE 3:**
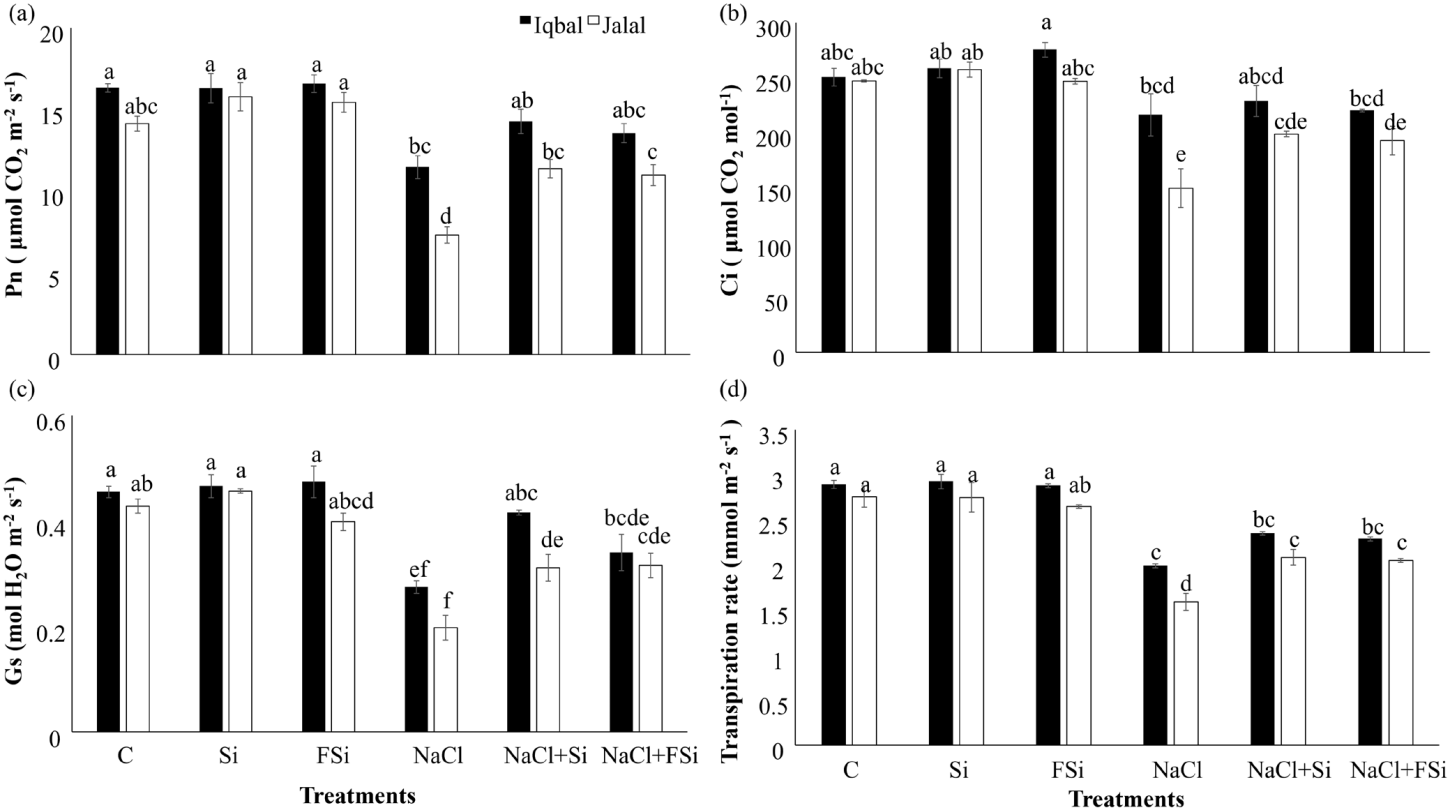
Effect of Ca_2_SiO_4_ on the growth of maize under salt stress. (a) Net photosynthesis (μmol CO_2_ m^−2^ s^−1^), (b) internal carbon (μmol CO_2_ mol^−1^), (c) stomatal conductance (mol H_2_O m^−2^ s^−1^), (d) transpiration rate (mmol m^−2^ s^−1^) in two different maize varieties (Iqbal and Jalal) under NaCl stress. The results were revealed in means, standard error (±), difference between the treatments (*p* ≤ 0.05, *n* ≥ 3) as indicated by different letters. (C): Control, (Si): 1 mM Ca_2_SiO_4_, (FSi): Foliar 1 mM Ca_2_SiO_4_, (NaCl) = 100 mM NaCl; (NaCl + Si) = 100 mM NaCl + 1 mM Ca_2_SiO_4_, (NaCl + FSi) = 100 mM NaCl + foliar 1 mM Ca_2_SiO_4_.

The effect of Ca_2_SiO_4_ on various gaseous exchange parameters, including net photosynthetic rate (*P*_n_), intercellular CO_2_ concentration (*C*_i_), transpiration rate (*T*_r_), and stomatal conductance (*G*_s_) on salt‐stressed plants is shown in Figure 3. Compared with controls, all parameters show strong negative responses to salt stress, Jalal more than Iqbal. In both varieties, *P*_n_ and *G*_s_ were more affected than Ci and Tr. Interestingly, Si treatment could not recover the salt stress on *G*_s_ and *T*_r_ (Figure 3).

### 3.3 Effect of Ca_2_SiO_4_ on Phytohormone Production in Salt‐Stressed Maize Varieties

**FIGURE 4:**
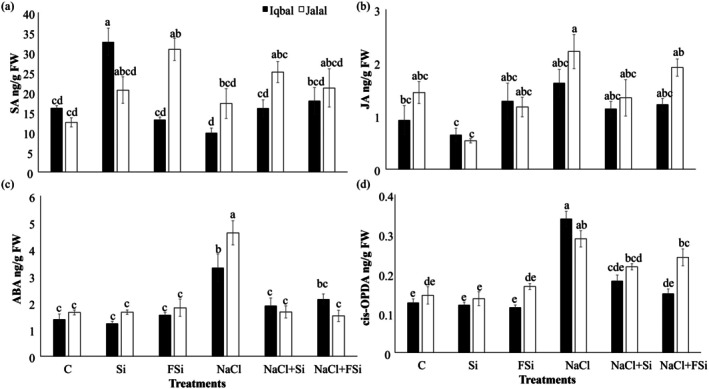
Effect of Ca_2_SiO_4_ on phytohormone production ng g^−1^ under salt stress. (a) Salicylic acid, (b) jasmonic acid, (c) abscisic acid, (d) jasmonoyl‐isoleucine in two different maize varieties (Iqbal and Jalal) under NaCl stress. The result was revealed in means, standard error (±), difference between the treatments (*p* ≤ 0.05, *n* ≥ 3) as indicated by different letters. (C): Control, (Si): 1 mM Ca_2_SiO_4_, (FSi): Foliar 1 mM Ca_2_SiO_4_, (NaCl) = 100 mM NaCl; (NaCl + Si) = 100 mM NaCl + 1 mM Ca_2_SiO_4_, (NaCl + FSi) = 100 mM NaCl + foliar 1 mM Ca_2_SiO_4_.

To get deeper insight in the endogenous regulation of the salt stress‐initiated responses and the attenuating effect of Si, we further analyzed the stress‐related phytohormone (salicylic acid [SA], jasmonic acid [JA], abscisic acid [ABA]) levels in the two maize varieties (Figure 4). In Iqbal and Jalal, the phytohormone levels in the control plants were similar. Strikingly, upon Si treatment alone, SA increased significantly in Iqbal by about 100%. In contrast, in Jalal, SA content increased significantly (*p* < 0.05) upon foliar Si treatment (Figure 4a). NaCl‐induced stress caused no significant changes, just a small decrease of SA in Iqbal compared to the control. Further Si treatment induced a higher but not significant SA level in Jalal (Figure 4a). JA level was clearly reduced by Si treatment in both varieties and slightly increased in Jalal upon NaCl stress; no more changes were detected (Figure 4b). The ABA concentration was noticed to be significantly higher under NaCl (*p* < 0.05) in both Iqbal and Jalal varieties compared to the control and almost all other treatments. However, a decline in ABA concentration with the introduction of Si with NaCl treatment was observed (Figure 4c). The biosynthetic JA precursor OPDA showed very similar results except that the decrease upon Si treatment was not detectable (Figure 4d).

### 3.4 Effect of Ca_2_SiO_4_ on Gene Expression in Salt Stressed Maize Varieties

**FIGURE 5:**
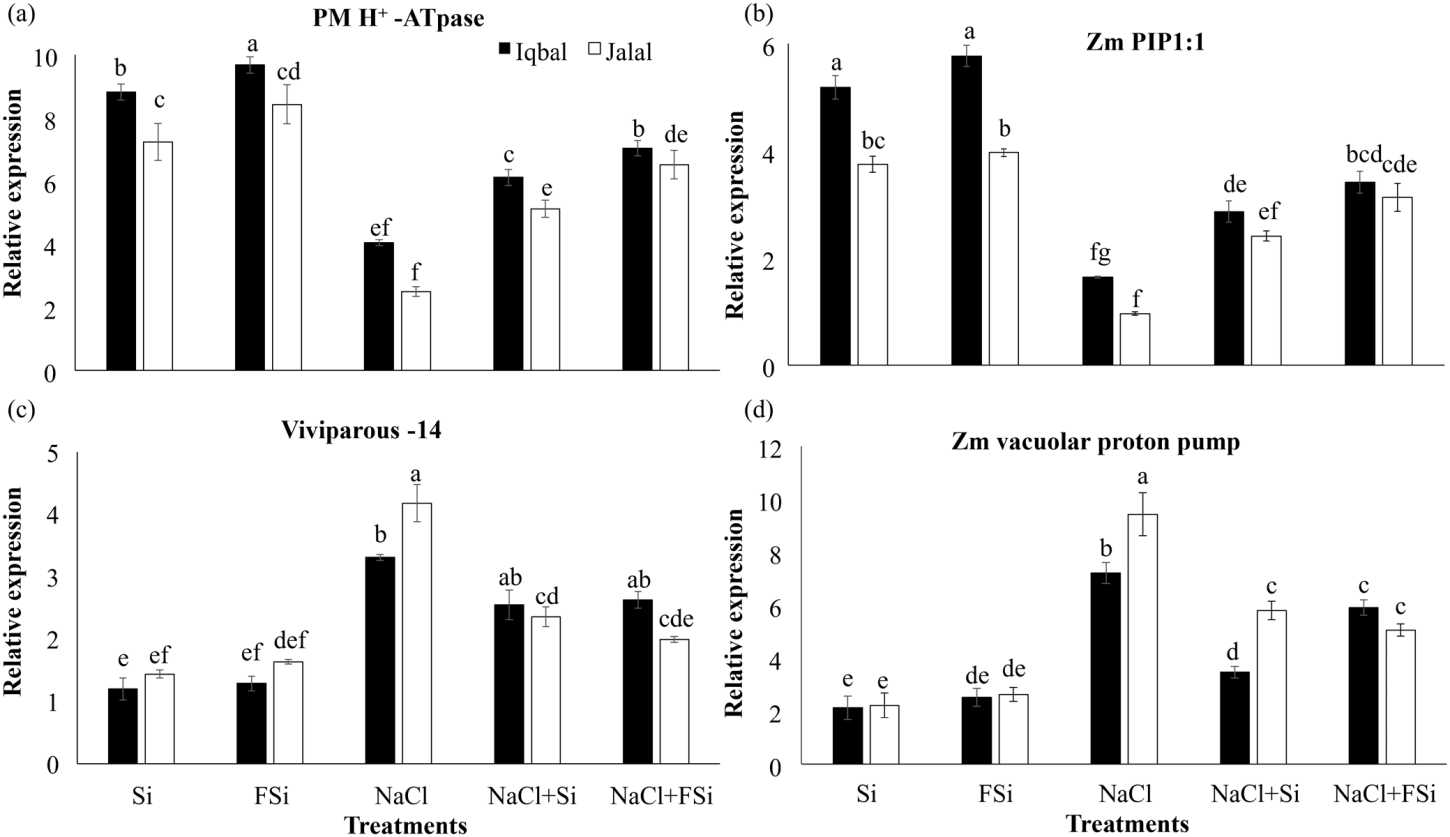
Effect of Ca_2_SiO_4_ on gene expression under salt stress. (a) Plasma membrane H^+^‐ATPases, (b) maize plasma membrane intrinsic protein, (c) maize Viviparous14, (d) vacuolar proton pump in two different maize varieties (Iqbal and Jalal) under NaCl stress. Untreated plants were used as controls and set to “1”. The result was revealed in means, standard error (±), difference between the treatments (*p* ≤ 0.05, *n* ≥ 3) as indicated by different letters. (Si): 1 mM Ca_2_SiO_4_, (FSi): Foliar 1 mM Ca_2_SiO_4_, (NaCl) = 100 mM NaCl; (NaCl + Si) = 100 mM NaCl + 1 mM Ca_2_SiO_4_, (NaCl + FSi) = 100 mM NaCl + foliar 1 mM Ca_2_SiO_4_.

Next, the impact of Si on the relative expressions of some key genes responding to salt stress was investigated and compared between the two varieties. This included genes encoding *PM H* ^+^ *‐ATPase*, *Zm PIP 1:1*, *VP‐14*, and *VPp3*. All treatments induced these genes; however, to different levels. In Jalal and Iqbal, *PM H* ^+^ *‐ATPase* was induced in the presence of Si. NaCl also induced this gene but about 50% less. In all combinations of Si and salt, the gene expressions were detected between these two values (Figure 5a). A very similar result was found for *PIP1:1* (Figure 5b). *VP‐14* and *VPp3* were induced in both varieties only by NaCl challenge more than 3‐ and 8‐fold, respectively, which was mitigated by Si (Figure 5c,d). Si alone had only minor effects.

### 3.5 Principal Component Analysis

**FIGURE 6:**
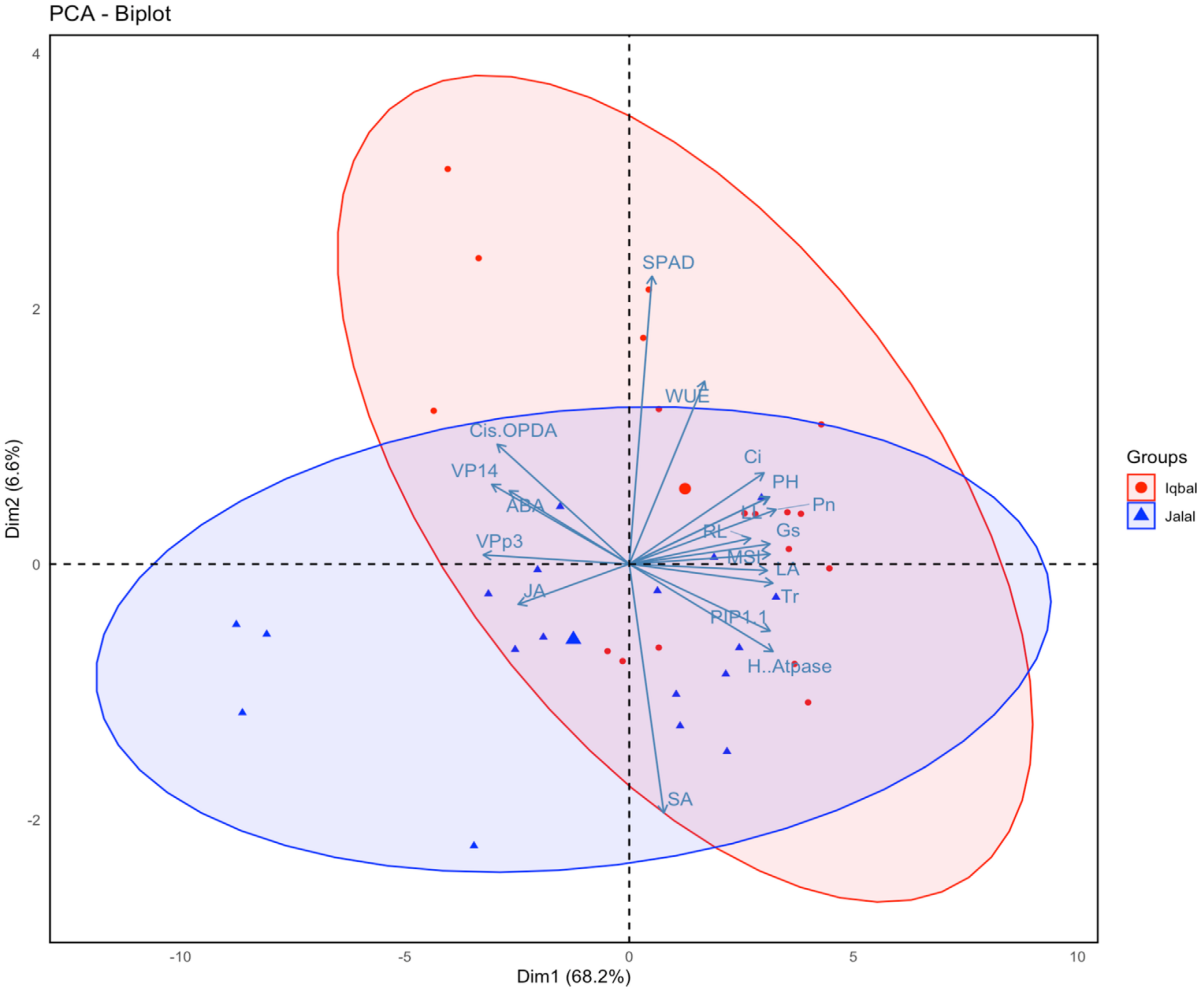
Principal component analysis (PCA) to explain the treatment–variable relationships in two maize varieties under both NaCl and Si treatment. The mean values obtained of different variables in this study were normalized and clustered. At the variable level, three distinct clusters (1–3) were identified. The abbreviations used in PCA of the data includes: FWL, fresh weight leaf; FWR, fresh root weight; PH, plant height, LL, leaf length; RL, root length; LA, leaf area; SPAD, chlorophyll content; *C*_i_, internal carbon; *P*_n_, photosynthetic rate; *T*_r_, transpiration rate; *G*_s_, stomatal conductance; MSI, membrane stability index; WUE, water use efficiency; SA, salicylic acid; JA, jasmonic acid; ABA, abscisic acid; *cis*‐OPDA, *cis*‐(+)‐12‐oxo‐phytodienoic acid; *PIP1:1*, plasma membrane instinct protein; *VPp3*, vacuolar proton pump; *VP14*, viviparous 14, and *H‐ATpase*.

A principal component analysis (PCA) was further used to identify key traits that might contribute to salt tolerance. Biplot analysis was used to visualize traits and treatments in a two‐dimensional space. The PCA score graph was done using the experimental dataset containing the two maize varieties and 19 variables to reduce data dimensionality and identify potential relationships among measured parameters (Figure 6). The principal components (PC) 1 and 2 explained 74.8% of the total variance of the dataset (Figure 6). In detail, the PCA results showed that the principal components of the Iqbal variety with eigenvalues of 13.37 accounted for 66.86% of the total variation, while the PCs of the Jalal variety with eigenvalues of 15.94 accounted for 79.73% variation. Samples of Iqbal and Jalal varieties formed distinct clusters and separated into two groups, which is evident along PC2. While Jalal samples mainly clustered in quadrants 3 (see big blue triangle) and 4, Iqbal samples clustered mainly in quadrants 1 (see big red circle) and 2, although few overlaps were found in all quadrants. According to correlation values of the variables with the PCs, this separation is mainly caused by the positive and negative correlation of PC2 with chlorophyll (SPAD) and WUE and SA, respectively, where the arrows indicate the strength of the trait influence on the two PCs (Figure 6).

**FIGURE 7:**
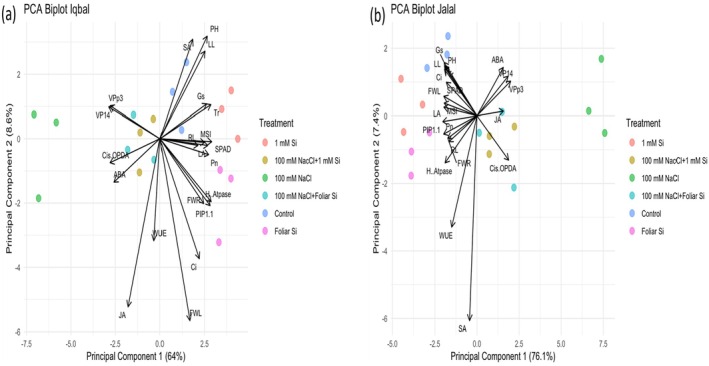
Principal component analysis (PCA) to explain the effect of Si on two maize varieties under salt stress (a) Iqbal (b) Jalal. The mean values obtained of different variables in this study were normalized and clustered. The abbreviations used in PCA of the data includes: FWL, fresh weight leaf; FWR, fresh root weight; PH, plant height, LL, leaf length; RL, root length; LA, leaf area; SPAD, chlorophyll content; *C*_i_, internal carbon; *P*_n_, photosynthetic rate; *T*_r_, transpiration rate; *G*_s_, stomatal conductance; MSI, membrane stability index; WUE, water use efficiency; SA, salicylic acid; JA, jasmonic acid; ABA, abscisic acid; *cis*‐OPDA, *cis*‐(+)‐12‐oxo‐phytodienoic acid; *PIP1:1*, plasma membrane instinct protein; *VPp3*, vacuolar proton pump; *VP14*, viviparous 14, and *H‐ATpase*. The lines originating from the central point of the biplots show negative or positive correlations of different variables, and their closeness indicates the strength of correlation with a particular treatment. (C): Control, (Si): 1 mM Ca_2_SiO_4_, (FSi): Foliar 1 mM Ca_2_SiO, (NaCl) = 100 mM NaCl; (NaCl + Si) = 100 mM NaCl + 1 mM Ca_2_SiO_4_, (NaCl + FSi) = 100 mM NaCl + foliar 1 mM Ca_2_SiO_4_.

In order to identify the individual effects of salt stress and Si treatment on the two varieties independently, additional PCA analyses were performed. In Iqbal, PC1 and PC2 explained 72.6% of the total variance (Figure 7a). Salt stress samples and Si‐treated samples are clearly separated from each other and from all other samples along PC1, while untreated controls and salt stress plus Si treatments cluster together. This separation is mainly driven by the negative correlation values of PC1 with VPp3, VP14, *cis*‐OPDA, JA, and ABA. In the Jalal variety, the PCA analysis showed almost the same results. PC1 and PC2 explained 83.5% of the total variance (Figure 7b). Salt stress samples are clearly separated from all other samples. Si‐treated samples cluster together with the control samples, and salt‐stressed plus Si‐treated samples also cluster together. Separation again is along PC1 and mainly due to the correlation values of PC1 with VPp3, VP14, *cis*‐OPDA, JA, and ABA on one side and all other parameters on the other side. Strikingly, again SA and all other phytohormone‐related features are opposing (Figure 7b).

## 4 Discussion

### 4.1 Salt Stress Effects on Maize Growth Parameters

Maize is a worldwide important crop that unfortunately is sensitive to high soil salinity, an increasing problem in many parts of the world. In this study, a salt sensitive (Jalal) and a salt tolerant (Iqbal) maize variety have been investigated concerning their response to Si (1mM Ca_2_SiO_4_) treatment under salt (100 mM NaCl) stress. In order to verify the expected salt sensitivity vs the salt tolerance in the two varieties, basic phenotypical parameters such as fresh weight (Figure 1), plant height, leaf length, and others (Figure 2) were determined in comparison to the untreated control plants. Almost all of these parameters showed that Iqbal was more tolerant of salt stress than Jalal. This was further supported by physiological analyses such as the MSI and WUE (Table 1), net photosynthesis rate, internal carbon content, or transpiration rate (Figure 3). The whole data set indicated that the Iqbal variety exhibited higher salt tolerance than Jalal, confirming recent results obtained by Mahmood et al. (2024). Several other studies have investigated the reduced biomass and other responses of plants to salt stress, encompassing various crops, including maize (Galić et al. 2020), wheat (Pour‐Aboughadareh et al. 2021), rice (Pires et al. 2015), soybean (Hamayun et al. 2010), and more. Understanding the mechanisms underlying the responses to salt stress is essential for developing strategies to mitigate its negative effects and enhance crop productivity under saline conditions (Riaz et al. 2019).

### 4.2 Effect of Ca_2_SiO_4_ Application on the Phenotypical Features in Salt Stressed Maize Varieties

Silicon influences cell expansion and differentiation. Applying silicon can stimulate nutrient uptake and transport, cell division and elongation, and promote the growth of shoots and roots (Mandlik et al. 2020). Bassiouni et al. (2020) observed a significant increase in the biomass of rice accompanied by reduced oxidative stress and enhanced antioxidant enzyme activities. Si deposition in plant cell walls contributes to their strength and rigidity, which enhances mechanical support for the plant, allowing it to withstand environmental stresses and maintain an upright posture. Additionally, strengthened cell walls can support greater cell expansion, leading to increased plant height and leaf area (Pierantoni 2019). Several studies have demonstrated the positive influence of Si on plant growth and biomass accumulation (El Sabagh et al. 2021; Mir et al. 2022; Seleiman et al. 2022). Gou et al. (2020) found that Si significantly increased plant biomass in cucumber by improving nutrient uptake and enhancing photosynthetic efficiency. Also, a significant increase in soybean biomass production following Si application showed improved root development and nutrient absorption (Attipoe et al. 2023). Si has been shown to mitigate the adverse effects of various abiotic stresses, such as salinity, acidity, and heavy metal toxicity. Our study is also in line with these studies, showing that the application of Ca_2_SiO_4_ significantly mitigated the salt stress effects and improved maize growth parameters in both varieties, independent of the way of Si application (Figures 1 and 2). Under both NaCl + Si and NaCl + FSi treatments, the tested phenotypical parameters recovered slightly better in the Jalal variety than in Iqbal (Figures 1 and 2). This might be due to the higher sensitivity of Jalal towards the salt stress.

All tested physiological responses (Figure 3) showed the same significant trends. While salt stress affected the studied plants' parameters, treatments with Si mitigated those effects (Figure 3). The analyzed gaseous exchange parameters, such as photosynthesis rate, stomatal conductance, transpiration rate, and intercellular CO_2_ concentration (Figure 3), are crucial indicators of plant physiological status and can provide insights into the plant's response to environmental stresses like salt stress. Studies demonstrated that Si can enhance the photosynthesis rate in crop plants subjected to salt stress. For instance, Al‐Huqail et al. (2019) reported that Si increased the photosynthesis rate in salt‐stressed crop plants by improving chlorophyll content, chlorophyll fluorescence parameters, and photosynthetic pigments. Souri et al. (2021) reported that Si treatment increased the photosynthesis rate in salt‐stressed plants by enhancing water and nutrient uptake. Si application has positively influenced plants' stomatal conductance and transpiration rate under salt stress conditions. Sattar et al. (2016) observed that Si‐treated plants exhibited higher stomatal conductance and transpiration rates than untreated plants under salt stress, indicating improved water use efficiency and reduced stomatal limitation. Limited studies have specifically investigated the effect of Ca_2_SiO_4_ on intercellular CO_2_ concentration in maize under salt stress. However, based on its positive impact on photosynthesis and stomatal conductance, it can be inferred that Si application may lead to a reduction in intercellular CO_2_ concentration in salt‐stressed maize plants, as higher photosynthesis rates and stomatal conductance would facilitate greater CO_2_ uptake and assimilation (Delavar et al. 2018).

Moreover, supplementation with Si caused higher chlorophyll content and MSI values in Si + NaCl and FSi + NaCl treatments compared to NaCl treatment alone (Table 2). WUE exhibited slight fluctuations across treatments, with generally higher values observed in Si‐treated plants than in NaCl‐treated ones. However, in these analyses, there were no clear significant differences between salt‐stressed and stressed and Si‐supplemented approaches. However, still, a clear explanation of how Si might act on the plant is missing.

### 4.3 Effect of Ca_2_SiO_4_ on Phytohormones Production in Salt Stressed Maize Varieties

Phytohormones are key regulators in plant physiology (Carlos et al. 2021). Si supplementation has been shown to modulate the levels of various phytohormones in maize through various mechanisms under stress conditions (Moradtalab et al. 2018). In the present study, Si application alone led to increased SA (depending on the mode of application) and decreased JA contents in both varieties, while ABA and OPDA contents remained unaffected (Figure 4). Strikingly, upon NaCl stress, the plants responded differently. Here ABA and jasmonates' contents were affected; ABA and OPDA increased significantly, while for JA only a trend was detected (Figure 4). These results suggest that Si and NaCl act antagonistically on the phytohormones. Consequently, the application of Si to salt‐stressed plants reduced the levels of ABA and OPDA, and the combination of Si and NaCl also reduced the levels of SA and JA almost to control levels (Figure 4). SA is a known key signaling molecule in plant defense responses against biotic and abiotic stresses, including pathogens and environmental stressors. Si induces SA biosynthesis by activating phenylalanine ammonia‐lyase and other enzymes in the phenylpropanoid pathway. SA accumulation triggers defense mechanisms, including the expression of pathogenesis‐related genes and the production of reactive oxygen species, to combat stress (Abdelaal et al. 2022). Jasmonates regulate plant defense responses, including insect resistance, wound healing, and abiotic stress tolerance. Si induces the expression of genes involved in JA biosynthesis, such as lipoxygenase and allene oxide synthase, leading to increased JA production. ABA is a crucial phytohormone in regulating plant responses to various environmental stresses, particularly drought and salinity. Si enhances ABA biosynthesis by upregulating the expression of genes encoding key enzymes in the ABA biosynthetic pathway, such as 9‐*cis*‐epoxycarotenoid dioxygenase. Increased ABA levels promote stomatal closure, osmotic adjustment, and the expression of stress‐responsive genes, thereby improving stress tolerance (Aslani et al. 2014).

### 4.4 Effect of Ca_2_SiO_4_ on Gene Expression in Salt Stressed Maize Varieties

The effect of Ca_2_SiO_4_ on gene expression in maize varieties under salt stress involves intricate molecular mechanisms that regulate stress‐responsive pathways, ion homeostasis, antioxidant defense, and other physiological processes (Khan et al. 2016). Ca_2_SiO_4_ treatment can induce salt stress‐responsive gene expression in signal transduction, osmotic adjustment, and detoxification pathways including transcription factors which regulate the expression of downstream stress‐related genes (Shah et al. 2021), as well as stress signaling cascades, including mitogen‐activated protein kinase pathways and calcium‐dependent protein kinases, leading to the phosphorylation and activation of transcription factors. Studying such genes can help get more insight into regulatory networks for plant survival and might identify intervention points for improving crop resilience to salinity. Compared with non‐treated controls, the expression of *PM H* ^+^ *‐ATPase* or *PIP1:1 genes was* clearly induced by Si treatment alone, in contrast to *VP14* and *VPp3P* genes. Strikingly, the two genes were much stronger induced by NaCl treatment than the genes *PM H* ^+^ *‐ATPase* and *PIP1:1* (Figure 5). Significant changes in gene expression were also observed when comparing NaCl treatment alone with NaCl combined with Si treatment. Under these conditions, the expression level of all four genes was found to be between the extremes, e.g., expression induced by Si or NaCl (Figure 5). Si treatment obviously can influence the expression of *PM H* ^+^ *‐ATPase* under salt stress conditions. Other studies have shown that NaCl can decrease gene expression levels of *PM H* ^+^ *‐ATPase* in crop plants (Olfatmiri et al. 2014). This suggests that Si may modulate ion transport mechanisms in response to salt stress. While specific studies on the effect of Ca_2_SiO_4_ on *ZmPIP1:1* gene expression are limited, it is reasonable that Ca_2_SiO_4_ supplementation may up‐regulate the expression of these genes to improve water balance in plants, which is affected under salt stress. The upregulation of the *VP14* gene under salt stress as well as its reduction in the presence of Si correlates perfectly with the increase of ABA (Figure 4) suggesting that the ABA accumulation depends on *de novo* synthesis. Our study showed that Ca_2_SiO_4_ supplementation can lead to changes in *VP14* gene expression, although the specific mechanisms involved require further investigation. While research on the specific effect of Si on *VPp3* gene expression is limited, it is credible that its supplementation may modulate *VPp3* gene expression to maintain ion balance and cellular homeostasis in plants. These findings contribute to the existing literature on salt stress mitigation strategies and the potential role of Ca_2_SiO_4_ in enhancing crop resilience to environmental stressors.

In this study, PCA was used to identify the most important selection traits for salt tolerance, and also to show how Si supplementation might help the plant to better grow in a saline environment (Figures 6 and 7a,b). PCA‐biplot is a multivariate analysis that combines traits and objects in two dimensions, minimizing overlapping variations, and aiding in identifying main characters for selection (Kose et al. 2018; Huqe et al. 2021). The PCA revealed that the traits chlorophyll content (SPAD), WUE, H^+^‐ATPase, and SA contributed strongly to discriminate between the two varieties, Iqbal and Jalal. The clustering patterns within the two varieties demonstrate a clear difference between salt stress and Si treatment. Strikingly, control and NaCl + Si treatments were found separated from the former two and clustered together, indicating that Si treatment can indeed mitigate the salt stress responses. This suggests that Si application in stress‐prone environments could be a viable agronomic strategy for improving crop productivity.

## Conflicts of Interest

The authors declare no conflicts of interest.

## Supporting information


Data S1–S3.

## Data Availability

The raw data that support the findings of this study are available in the Supporting Information of this article.
