# Supplementary material for: Silicon‐Mediated Mitigation of Salt Stress in Maize Plants
Source: Plant Environ Interact. 2025 Jul 28;6(4):e70073. doi: 10.1002/pei3.70073 (PMC12302281; doi:10.1002/pei3.70073)
Supplement: Supplementary file 1 — Data S1–S3. [file PEI3-6-e70073-s001.zip › pei370073-sup-0003-DataS3.docx]

|  | 1. [Zea mays vacuolar: ATPase subunit H protein (LOC100280574), mRNA](https://blast.ncbi.nlm.nih.gov/Blast.cgi#alnHdr_2171723323) |
| --- | --- |
|  | Accession number NM_001153493.2 |

|  | Sequence (5'->3') | Template strand | Length | Start | Stop | Tm | GC% | Self complementarity | Self 3' complementarity |  |
| --- | --- | --- | --- | --- | --- | --- | --- | --- | --- | --- |
| Forward primer | CCGTCTCGCCCTCATCTAA | Plus | 19 | 2 | 20 | 58.58 | 57.89 | 2.00 | 1.00 |  |
| Reverse primer | TGGAAGAAGGATCGGGAGAC | Minus | 20 | 112 | 93 | 58.51 | 55.00 | 4.00 | 1.00 |  |
| Product length | 111 | | | | | | | | | |

Fwd self -3.61 kcal/mol  Base Pairs:  2

Rev self -4.62 kcal/mol  Base Pairs:  4

Heterodimer  -6.68 kcal/mol   Base Pairs:  3

1. **Zea mays vacuolar proton pump 3 (LOC542327), mRNA**

Accession number: NM_001319777.2

|  | Sequence (5'->3') | Template strand | Length | Start | Stop | Tm | GC% | Self complementarity | Self 3' complementarity |
| --- | --- | --- | --- | --- | --- | --- | --- | --- | --- |
| **Forward primer** | GCACGGTGAACTGCTGTAGA | Plus | 20 | 2024 | 2043 | 60.04 | 60.00 | 5.00 | 2.00 |
| **Reverse primer** | AGAAATGAGTAGCGGTGGGG | Minus | 20 | 2142 | 2123 | 59.46 | 55.00 | 2.00 | 0.00 |
| **Product length** | 118 (mix) | | | | | | | | |

Fwd self  -5.09 kcal/mol  Base Pairs:  3

Rev self   -3.61 kcal/mol  Base Pairs:  2

Heterodimer  -4.74 kcal/mol   Base Pairs:  3

1. **Zea mays plasma membrane intrinsic protein (LOC542619), mRNA**

Accession number: NM_001112146.1

|  | Sequence (5'->3') | Template strand | Length | Start | Stop | Tm | GC% | Self complementarity | Self 3' complementarity |  |
| --- | --- | --- | --- | --- | --- | --- | --- | --- | --- | --- |
| Forward primer | CGGAGGCGTGAACTGTAGAT | Plus | 20 | 947 | 966 | 59.54 | 55.00 | 3.00 | 2.00 | |
| Reverse primer | ATGGCTAGAGGCAACCAACG | Minus | 20 | 1018 | 999 | 60.39 | 55.00 | 4.00 | 2.00 | |
| Product length | 72 | | | | | | | | | |

|  | Fwd self -3.61 kcal/mol  Base Pairs:  2  Rev self -5.02 kcal/mol  Base Pairs:  3  Heterodimer -4.95 kcal/mol   Base Pairs:  3 |
| --- | --- |

1. **Zea mays viviparous14 (LOC732819), ZmVp14**

Accession number: NM_001112432.3

|  | Sequence (5'->3') | Template strand | Length | Start | Stop | Tm | GC% | Self complementarity | Self 3' complementarity |
| --- | --- | --- | --- | --- | --- | --- | --- | --- | --- |
| Forward primer | TGTTGTCACCCAGTCCAGTG | Plus | 20 | 2303 | 2322 | 59.82 | 55.00 | 3.00 | 3.00 |
| Reverse primer | CCGATAGCCACAGGGAACAC | Minus | 20 | 2443 | 2424 | 60.46 | 60.00 | 2.00 | 0.00 |
| Product length | 141 | | | | | | | | |

Fwd self:   -3.61 kcal/mol  Base Pairs:  2

Rev self:  -3.61 kcal/mol  Base Pairs:  2

Heterodimer:   -3.61 kcal/mol   Base Pairs:  2
